# Coactivation of Lower Limb Muscles during Gait in Patients with Multiple Sclerosis

**DOI:** 10.1371/journal.pone.0158267

**Published:** 2016-06-23

**Authors:** Julien Boudarham, Sophie Hameau, Raphael Zory, Alexandre Hardy, Djamel Bensmail, Nicolas Roche

**Affiliations:** 1 INSERM 1179, CIC 1429, CHU Raymond Poincaré, APHP, University of Versailles Saint Quentin en Yvelines, Garches, France; 2 LAMHESS, EA 6312, University of Nice Sophia Antipolis, Nice, France; University Hospital, SWITZERLAND

## Abstract

**Background:**

Coactivation of agonist and antagonist lower limb muscles during gait stiffens joints and ensures stability. In patients with multiple sclerosis, coactivation of lower limb muscles might be a compensatory mechanism to cope with impairments of balance and gait.

**Objective:**

The aim of this study was to assess coactivation of agonist and antagonist muscles at the knee and ankle joints during gait in patients with multiple sclerosis, and to evaluate the relationship between muscle coactivation and disability, gait performance, dynamic ankle strength measured during gait, and postural stability.

**Methods:**

The magnitude and duration of coactivation of agonist-antagonist muscle pairs at the knee and ankle were determined for both lower limbs (more and less-affected) in 14 patients with multiple sclerosis and 11 healthy subjects walking at a spontaneous speed, using 3D-gait analysis.

**Results:**

In the patient group, coactivation was increased in the knee muscles during single support (proximal strategy) and in the ankle muscles during double support (distal strategy). The magnitude of coactivation was highest in the patients with the slowest gait, the greatest motor impairment and the most instability.

**Conclusion:**

Increased muscle coactivation is likely a compensatory mechanism to limit the number of degrees of freedom during gait in patients with multiple sclerosis, particularly when postural stability is impaired.

## Introduction

Normal motor control involves the coactivation of agonist and antagonist muscles. During gait, joint stiffness [1] and postural stability [2] are regulated by variations in the forces produced by the simultaneous contraction of antagonistic lower limb muscles. However, the functional role of coactivation remains unclear. Inappropriate coactivation (excessive and/or prolonged) reduces gait performance by reducing gait speed and increasing metabolic cost [1,3].

Increased coactivation of agonist and antagonist muscles at the knee and/or ankle joints during gait is a frequent problem in patients with central nervous system lesions caused by cerebral palsy [4], stroke and traumatic brain injury [5,6], Parkinson’s disease [7] and cerebellar ataxia [8]. Although the patterns of coactivation differ between these pathologies, the consequences are similar. Excessive coactivation of ankle muscles in the less-affected (LA) lower limb during the double support phase (DS), the initial (DS1) and the final double support (DS2), has been reported in patients with hemiparesis. This seems to reflect an adaptive, compensatory strategy to ensure postural stability, despite the loss of coactivation in the more-affected (MA) lower limb [5]. Similarly, excessive coactivation of the knee and ankle flexors and extensors has been found during stance phase in both lower limbs in patients with cerebral palsy [4] and cerebellar ataxia [8]. These patients also lack postural stability during gait. This loss of stability has been related to a lack of activation and reduced force output of the ankle plantarflexor muscles during gait [9]. Reduced ankle plantarflexor strength could limit safe weight transfer during the DS phases (DS1 and DS2) and bodyweight support during the single support (SS) phase. Increased muscle coactivation could therefore compensate for the weakness of the ankle plantarflexor muscles, increasing stability in stance [9,10].

Multiple sclerosis (MS) is a progressive neurological disease which occurs predominantly in young adults and causes demyelination of nerve fibers in the central nervous system [11]. Although symptoms vary considerably, cognitive, sensory and motor impairments are common [12]. Motor impairments include loss of selective muscle control, muscle weakness, abnormal muscle tone, ataxia and fatigue. These impairments often lead to loss of balance and gait performance [13] which are reported by patients as being the most disabling consequences of the disease [14]. The gait pattern of patients with MS is characterized by a large variability of spatial and temporal parameters across gait cycles [13,15], strongly associated with postural instability and an increased risk of falls [16]. Gait speed, step length, range of hip, knee and ankle motion and propulsive force are all reduced [17,18]. Few studies have evaluated the neuromuscular strategies adopted by these patients to cope with their impairments. In order to determine appropriate treatments to improve gait, it is important to understand how the loss of stability which frequently occurs in MS [19] affects gait, and if muscle coactivation at the knee and the ankle joints could compensate for a loss of stability. This question is particularly relevant since both lower limbs are often affected, thus between-limb compensation may be limited. However, to our knowledge, no study has until now investigated coactivation of lower limb muscles during gait in patients with MS.

Therefore, the first aim of this study was to determine the magnitude and duration of coactivation of agonist and antagonist muscles at the knee and ankle joints during gait in patients with MS. We hypothesized that muscle coactivation would be increased in both lower limbs (MA and LA) during all sub-phases of stance compared to healthy subjects. In order to increase understanding of the function of coactivation in these patients, the second aim was to evaluate the relationship between muscle coactivation during gait and disability, gait performance, ankle plantarflexor muscle strength and postural stability.

## Materials and Methods

### Participants

Fourteen patients with MS were enrolled in the study. The inclusion criteria were: over 18 years old, Expanded Disability Status Scale (EDSS) score of 5 or less (disability severe enough to impair full daily activities and ability to work a full day without special provisions. Patient able to walk without aid or rest for 200 m) [20], no changes in medication for relapse during the 30 days before participation in the study, no botulinum toxin injections in the lower limb muscles within the previous 4 months, no neurosurgery within the previous 6 months. Eleven healthy subjects matched for age and sex with no neurological or musculoskeletal pathologies were also enrolled as control subjects. This study was approved by the local ethics committee, “Comité de Protection des Personnes Ile de France XI”, and all subjects provided written informed consent prior to participation in any study-specific procedures.

### Experimental setup

#### Clinical exam

Spasticity of the quadriceps, hamstring and triceps surae muscles was evaluated using the Modified Ashworth Scale (MAS) [21]. Hip, knee and ankle flexor/extensor muscle strength was evaluated using the Medical Research Council (MRC) scale. MRC scores of the six muscle groups were averaged and summed for each lower limb. This score was used to define the MA and the LA lower limbs [22]. EDSS score was also determined [20].

#### Gait analysis

Gait parameters were recorded using 8 optoelectronic cameras (Motion Analysis, CA, USA, sampling frequency 100Hz) which measured the coordinates of 30 reflective markers. Markers were positioned according to the Helen Hays protocol. They were then filtered using a fourth-order zero-lag Butterworth low-pass-filter, with a 6 Hz cut off frequency [23]. Each gait trial was carried out in a 10 m gait corridor (6 gait trials). This corridor allowed at least eight successive gait cycles to be recorded. Each subject (healthy and patient) walked at their self-selected velocity and wore the same training shoes, to ensure reliable comparisons. Ground reaction forces were measured synchronously using two force plates (AMTI, Watertown, MA, USA, sampling frequency 1000 Hz) staggered along the walkway. Spatiotemporal parameters and joint moments were calculated for each gait cycle, using OrthoTrack 6.5 software (Motion Analysis Corporation, Santa Rosa, CA, USA). Inverse kinetics calculations were carried out on the kinetic data (Grood and Suntay method) [24] with Dempster's anthropometric table [25].

The spatiotemporal parameters calculated were: gait velocity, step length, cadence and step width. In addition, the duration of single support (SS) phase for each lower limb, the duration of initial (DS1) and final double support phase (DS2), the coefficients of variation (CV) of the entire cycle duration, step length and step width were also computed, since these parameters are known to be related to stability during gait in patients with MS [26].

In order to determine the relationship between ankle plantarflexor muscle strength and muscle coactivation at the knee and ankle joints, the peak ankle plantarflexor moment was computed for each lower limb during stance, as an index of dynamic ankle strength in a functional condition, as proposed by Lamontagne et al [2].

#### Electromyographic assessment

Surface electromyographic (sEMG) activity of the Rectus Femoris (RF), Vastus Lateralis (VL), Biceps Femoris (BF), Medial Gastrocnemius (MG), Soleus (SOL) and Tibialis Anterior (TA) was recorded during the gait trials. Six bipolar surface electrodes (Motion Lab Systems, LA, USA) were placed directly on the skin [27]. The sEMG sensors were composed of two circular dry button electrodes with double-differential preamplifiers. The active electrodes measured 12mm in diameter and the inter-electrode distance was 17mm. All sEMG signals were sampled at 1000Hz, pre-amplified with an amplification factor of 20. Before processing the EMG signal, all signals were band-pass filtered between 3.5 and 350Hz. The raw sEMG signals from each muscle were then time-normalized to 1000 points, corresponding to a gait cycle from 0 to 100% with 0.1% increments.

The method used by Chow et al., (2012) [6] was used to assess muscle coactivation during gait. A recent systematic review [28] indicated that this method is among the most adapted methods for this purpose. The coactivation indexes (CoI) and the coactivation durations (CoD) of RF and BF, VL and BF, TA and MG, and TA and SOL were computed during the gait cycle [6].

The CoI was calculated by dividing the area of overlap of the magnitude-normalized EMG of the agonist and antagonist muscles, by the duration of the overlap.

The CoD was calculated as the duration of overlap of activity of muscle pairs, computed using a Teager—Kaiser energy operator [29] and expressed as a percentage of the phase duration.

The CoI and CoD were calculated for both lower limbs (MA and LA) of the patients with MS. In the healthy subjects, the mean of the lower limbs was calculated for each. This analysis was performed for the stance phase (ST), the swing phase (SW) and during each sub-phase of stance: initial and final double support (DS1 and DS2 respectively) and SS. The analysis of the different sub-phases of the gait cycle provides information regarding the time course of changes in coactivation. During DS1 and DS2, both lower limbs are in contact with the ground and the body weight is transferred from one limb to the other. During SS, only one limb is in contact with the ground while the opposite limb is in swing phase.

### Statistics

Values from the two clinical evaluation scales (MAS and MRC) are expressed as medians. Values from the spatiotemporal and coactivation parameters are expressed as means ± standard deviation (SD).

The Wilcoxon test was used to compare clinical parameters (spasticity and strength) between the MA and LA lower limbs within each patient with MS and to compare the spatiotemporal gait parameters (velocity, cadence and step width and CV of step width) between patients and healthy subjects.

For the other spatiotemporal gait parameters (step length, single and DS duration, CV of step length and CV of cycle duration) and for each coactivation parameter (CoI and CoD), a Kruskall—Wallis test was used to analyze differences between the MA, LA and healthy limbs. Post-hoc comparisons were performed with the Mann—Whitney U-test, with the threshold of significance fixed at p<0.05.

Spearman’s correlations were performed in order to determine the relationship between the coactivation parameters (CoI and CoD of the MA and LA limbs) and level of disability (EDSS score and MAS values), gait performance (mean gait speed), dynamic ankle strength (peak ankle plantarflexor moment) and gait stability (mean single and DS duration and mean CV of the entire cycle duration, step length and step width). Moreover, Spearman’s correlations were performed between the EDSS score and the MAS values for each muscle tested, for both the MA and the LA lower limb, in order to evaluate the relationship between spasticity and the neurological impairment. Significance was set at p<0.012, following Bonferroni correction for the 4 groups of comparisons (disability, gait performance, dynamic ankle strength and postural stability).

All statistical analyses were performed using Statistica 7 (StatSoft, Tulsa, OK, USA).

## Results

The characteristics of both groups and the results of clinical examinations are presented in Tables 1 and 2 respectively.

**Table 1:** Demographic characteristics of the subjects.

Group characteristics		
	Patients with MS (N = 14)	Healthy subjects (N = 11)
Gender (M/F)	8/6	8/3
Age (years)	51 (12)	44 (14)
Height (cm)	171 (7)	175 (11)
Weight (kg)	68 (14)	77 (17)
EDSS	3.8 [2.6,4.4]	-

Mean (SD) values are presented for demographic characteristics. Median values [1st; 3rd quartiles] are presented for EDSS scores. EDSS = Expended Disability Status Scale, F = female, L = left, M = male, R = right.

**Table 2:** Results of the clinical examination for the patients with MS.

Clinical examination			
		Patients with MS (N = 14)	
Spasticity		MA lower limb	LA lower limb
MAS	Quadriceps	0[0,1]	0[0,0] #
	Hamstrings	0[0,1]	0[0,0]
	Triceps surae	1[0,1]	1[0.3,1]
**Strength**			
MRC scale	Sum	22.0	26.8 #
	Hip extensors	4[3.1,4.4]	4.5[4,5] #
	Hip flexors	3.5[3,4]	5[4,5] #
	Knee extensors	4[4,4.9]	5[5,5] #
	Knee flexors	3.8[3,4]	4[4,5] #
	Ankle dorsiflexors	4[3,4]	5[4,5] #
	Ankle plantarflexors	4[2.3,4]	4.5[4,5] #

Median values [1st; 3rd quartiles] are presented. EDSS = Expanded Disability Status Scale, LA = least-affected, MA = most-affected, MAS = Modified Ashworth Scale, MRC = Medical Research Council.

^#^ Significant difference between MA and LA limbs (p<0.05)

### Spatiotemporal parameters (Table 3)

**Table 3:** Results of spatiotemporal and kinetic gait parameters.

Spatiotemporal parameters			
	Patients with MS (N = 14)		Healthy subjects (N = 11)
	MA lower limb	LA lower limb	
Velocity (m/s)	0.87 (0.21) **		1.32 (0.22)
Step length (m)	0.51 (0.11) **	0.51 (0.08) **	0.67 (0.06)
Cadence (step/min)	100.1 (13.6) **		116.0 (10.1)
Step width (m)	0.17 (0.04)		0.16 (0.02)
Single support (%)	33.2 (2.7) **	35.7 (2.0) **#	40.8 (1.4)
Initial double support (%)	15.4 (1.9) **		8.0 (2.3)
Final double support (%)	15.7 (2.1) **		9.2 (2.6)
CV step length (%)	7.4 (2.7) **	6.3 (1.9) **	4.5 (1.8)
CV cycle duration (%)	4.6 (2.0) *		2.9 (1.1)
CV step width (%)	12.7 (9.0)		10.1 (4.4)

Mean (SD) values are presented. CV = coefficient of variation, LA = least-affected, MA = most-affected.

* Significant difference between patients with MS and healthy subjects (p<0.05)

** Significant difference between patients with MS and healthy subjects (p<0.001)

^#^ Significant difference between MA and LA limbs (p<0.05)

Compared to the healthy subjects, gait velocity and cadence were lower and step length and SS duration were shorter in the patients with MS (p<0.001). Initial (DS1) and final double support (DS2) duration were longer (p<0.001) in the patients with MS compared to the healthy subjects. CV of step length (p<0.001) and CV of cycle duration (p<0.05) were higher in the patient group.

In the patient group, SS duration (p<0.001) was longer in the LA lower limb compared to the MA lower limb.

### Coactivation parameters (Fig 1)

**Fig 1:**
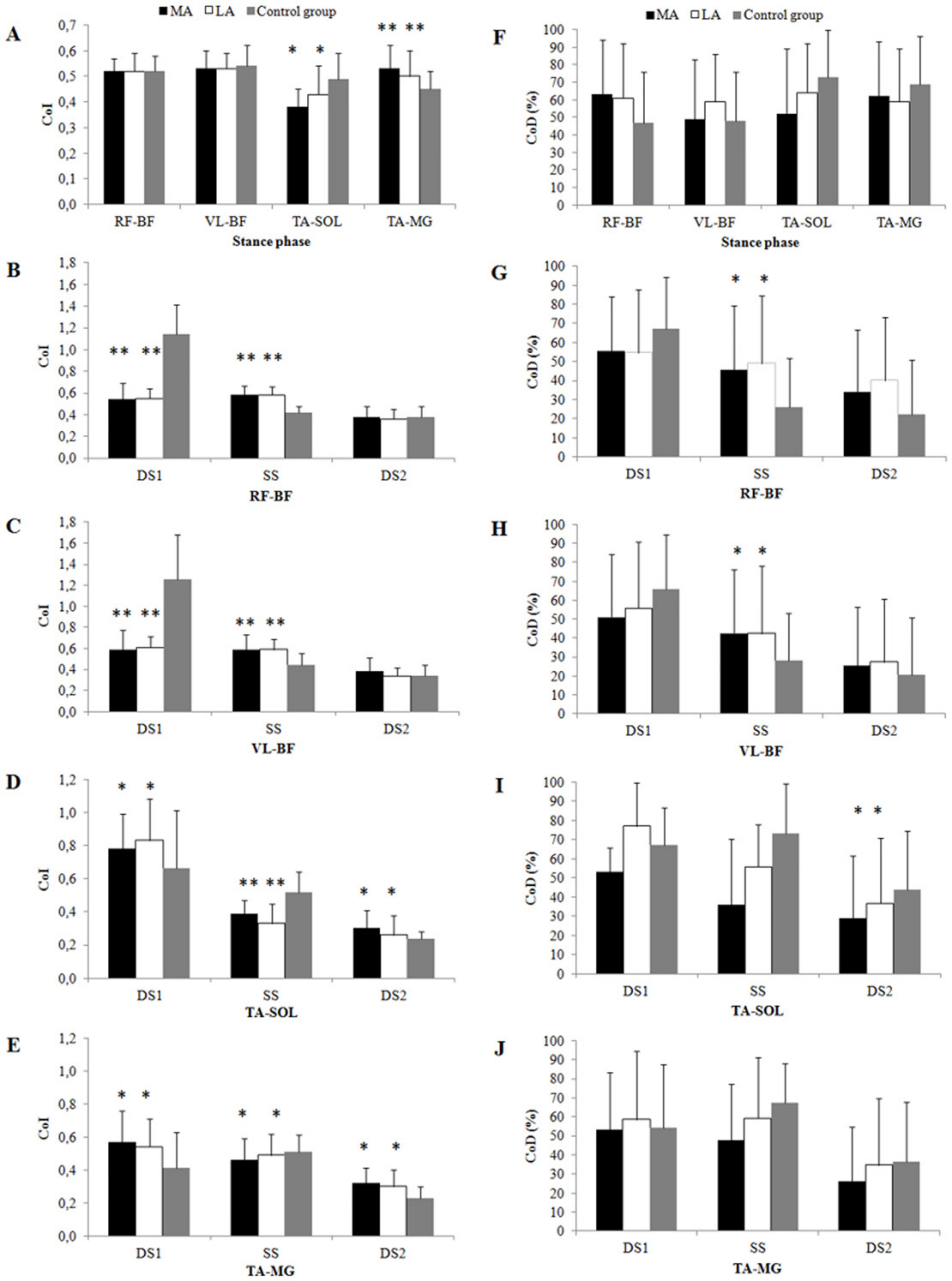
Mean and standard deviations (error bars) of the coactivation index (left panel) and coactivation duration (right panel) during total stance (A and F) and sub-phases of stance for RF-BF (B and G), RF-VL (C and H), TA-SOL (D and I) and TA-MG (E and J). LA = less-affected limb; MA = more-affected limb, DS1 = initial double support, SS = single support, DS2 = final double support, RF-BF = coactivation of rectus femoris and biceps femoris, VL-BF = coactivation of vastus lateralis and biceps femoris, TA-SOL = coactivation of tibialis anterior and soleus, TA-MG = coactivation of tibialis anterior and medial gastrocnemius. * Significant difference between patients with MS and healthy subjects (p<0.05). ** Significant difference between patients with MS and healthy subjects (p<0.001).

**CoI**.

***Knee muscles***. Compared to the healthy subjects:

- During DS1: the CoIs of i) RF-BF, and ii) VL-BF were lower (p<0.01) for both lower limbs of the patients with MS.
- During SS: the CoIs of i) RF-BF, and ii) VL-BF were higher (p<0.01) for both lower limbs of the patients with MS.

In the patients with MS:

- There were no significant differences between the MA and LA limbs during any of the sub-phases of the gait cycle.

***Ankle muscles***. Compared to the healthy subjects:

- During ST: the CoI of TA-MG was significantly lower (p<0.05) for both lower limbs of the patients with MS.
- During DS1: the CoI of i) TA-SOL, and ii) tibialis TA-MG were significantly higher (p<0.05) for both lower limbs of the patients with MS.
- During SS: the CoI of i) TA-SOL, and ii) TA-MG were significantly lower (p<0.05) for both lower limbs of the patients with MS.
- During DS2: the CoI of i) TA-SOL and ii) TA-MG were significantly higher (p<0.05) for both lower limbs of the patients with MS.

In the patients with MS:

- There were no significant differences between the MA and LA limbs during any of the sub-phases of the gait cycle.

**CoD**.

***Knee muscles***. Compared to the healthy subjects:

- During SS: the CoD of i) RF-BF, and ii) VL-BF were longer (p<0.05) for both lower limbs of the patients with MS.
- During DS2: the CoD of TA-SOL was significantly shorter (p<0.05) for both lower limbs of the patients with MS.

In the patients with MS:

- There were no significant differences between the MA and LA limbs during any of the sub-phases of the gait cycle.

***Ankle muscles***. There were no significant differences between the MS group and the healthy subjects during any of the sub-phases of the gait cycle.

There were no significant differences between the MA and LA limbs during any of the sub-phases of the gait cycle.

**Correlation**.

***Disability***. There were no significant correlations between EDSS score and the CoI or CoD of either the MA or LA limbs. Moreover, there were no significant correlations between the MAS values for each muscle tested (quadriceps, hamstring and triceps surae) and the CoI or CoD, in either the MA or LA limbs. In addition, it can be noticed that there were no significant correlations between the MAS values for each muscle tested (quadriceps, hamstring and triceps surae), of both MA and LA limbs, and the EDSS score.

***Gait performance***. For the MA lower limb:

- During DS2, the CoI of TA-MG was significantly negatively correlated with gait speed (ρ = -0.718, p = 0.003). This indicates that patients with an increased CoI at the ankle in the MA limb during DS2 were the slowest walkers.

For the LA lower limb:

- There were no significant correlations between gait speed and CoI or CoD.

***Dynamic ankle strength***. For the MA lower limb:

- During SS, the CoI of TA-MG was positively correlated with the peak ankle plantarflexor moment (ρ = 0.842, p = 0.002). This indicates that patients with an increased CoI at the ankle in the MA limb during SS had the highest dynamic strength of the ankle plantarflexors.
- During DS2, the CoI of TA-SOL was negatively correlated with the peak ankle plantarflexor moment (ρ = -0.757, p = 0.011). This indicates that patients with an increased CoI at the ankle in the MA limb during DS2 had the lowest dynamic strength of the ankle plantarflexors.

For the LA lower limb:

- There were no correlations between peak ankle plantarflexor moment and CoI or CoD.

***Postural stability***. For the MA lower limb:

- During SS, the CoI of i) RF-BF, and ii) VL-BF were significantly positively correlated with the CV of the cycle duration (ρ = 0.762, p = 0.001 and ρ = 0.674, p = 0.008, respectively). The CoI of TA-SOL was significantly positively correlated with SS duration (ρ = 0.731, p = 0.003). This indicates that patients with an increased CoI at the ankle and the knee in the MA limb during SS had the most unstable gait.
- During DS2, the CoI of TA-MG was positively correlated with the CV of the cycle duration (ρ = 0.727, p = 0.003). This indicates that patients with an increased CoI at the ankle in the MA lower limb during DS2 had the most unstable gait.

For the LA lower limb:

- During DS1, the CoI of TA-SOL was negatively correlated with the CV of step length and the CV of the cycle duration (ρ = -0.652, p = 0.011 and ρ = -0.780, p = 0.009, respectively). This indicates that patients with an increased CoI at the ankle in the LA lower limb during DS1 had the most stable gait.

## Discussion

To our knowledge, this study is the first to measure coactivation of agonist-antagonist muscles at the knee and ankle during gait in patients with MS and to compare values with healthy subjects. The key finding was that muscle coactivation differed in the patients with MS compared to the heathy subjects, depending on the sub-phase of stance. Moreover, there were no differences between coactivation in the MA and the LA limbs. In addition, we found that the patients who exhibited the highest level of muscle coactivation in the MA lower limb: i) were the slowest walkers; ii) had the greatest dynamic plantarflexor weakness and iii) were the most unstable during gait.

### Muscle coactivation during double support

There was excessive coactivation in the agonist and antagonist ankle muscles of the patients with MS during DS1 and DS2. Higher cycle-to-cycle variability, prolonged duration of the DS sub-phases (DS1 and DS2) and reduced gait velocity have been reported in patients with MS [13,15], and related to an impairment of dynamic balance control [26,30]. Thus excessive coactivation around the ankle during double support could be related to a compensatory strategy adopted by the neuromuscular system to improve mechanical stability [1,2,3,5]. During the DS phase, increased coactivation stiffens joints, reducing degrees of freedom [1,2], and could improve the efficiency of weight acceptance (during DS1) and weight transfer to the future supporting limb (during DS2) [8]. The present results support this hypothesis and also indicate that this strategy particularly involves the MA lower limb. The increased CoI of TA-MG and TA-SOL of the MA limb were positively correlated with the CV of the cycle duration during DS2 and SS respectively, reflecting postural instability during gait [13]. The patients with the most postural instability had the highest levels of coactivation around the ankle in the MA limb. In contrast, the CoI of TA-SOL of the LA limb was negatively correlated with the CV of step length and the CV of cycle duration, indicating that patients with increased ankle coactivation in the LA limb had the most stable gait. This suggests that coactivation of ankle muscles of the MA limb plays a key role in the control of postural stability during gait, in contrast with the LA limb. Moreover, the patients with the lowest dynamic plantarflexor strength in the MA limb (lowest peak ankle plantarflexor moment) had the greatest magnitude of ankle muscle coactivation during DS2. Since weakness of the ankle muscles is related to loss of stability [31], the increased coactivation of the ankle muscles in stance phase could be a mechanism to compensate for weakness in the most unstable patients.

A second hypothesis is that the excessive ankle muscle coactivation during double support is caused by spasticity. Spasticity-related muscle over-activity could increase the magnitude and the duration of the sEMG signals recorded for MG and SOL, increasing the values of the coactivation parameters (CoI and CoD) at the ankle. Other authors have previously proposed that spasticity of the ankle plantarflexors is the cause of increased muscle over-activity (in intensity and duration) during gait in patients with MS [17]. In contrast with the literature, the results of our study did not show any correlation between the MAS values of any muscle and the EDSS scores in either the MA or LA limb [32]. This must, however be interpreted with caution since the patients included had low levels of spasticity, MAS score (tested statically) many not reflect spasticity during dynamic gait [33] and the modified Ashworth Scale has been shown to be unreliable [34]. The most pertinent method to assess the impact of spasticity during gait is to use a musculo-skeletal model. Further study using such model would be pertinent to accurately evaluate the relationship between muscle coactivation parameters and spasticity.

Taken together, these results strongly suggest that during the DS phases, patients with MS mainly use a “distal” strategy, involving increased ankle muscle coactivation in the MA lower limb in order to stiffen the ankle joint and improve the stability of weight acceptance and the efficiency of weight transfer. These results are, moreover, in accordance with previous results in the literature which suggest that increased coactivation of ankle muscles is an adaptive strategy [5,6,8,26].

Finally, excessive ankle muscle coactivation in the MA limb during DS2 was associated with slow gait. Increased muscle coactivation may increase joint stiffness, decreasing the peak ankle plantarflexion moment [23] and limiting gait speed [35]. However, it is unlikely that this was the only cause of slow gait in the patients in the present study. Firstly, in healthy subjects, it has been shown that increased muscle coactivation increases gait speed [3,36]. Secondly, in older subjects and patients with neurological impairments, slow gait is considered to be a compensatory strategy to improve postural stability and limit falls [37,38,39]. It thus appears that in patients with MS, the neuromuscular system simultaneously reduces gait speed and increases ankle muscle coactivation to ensure stability.

### Muscle coactivation during single support

During the SS sub-phase of stance, coactivation of the ankle muscles was reduced (lower CoI of TA-SOL and TA-MG) and coactivation of the knee muscles was increased (higher CoI and longer CoD of RF-BF and VL-BF) in the patients with MS. The reason for the excessive coactivation of the thigh muscles during SS is unclear. Firstly, it could be a mechanism to compensate for “insufficient” ankle muscle coactivation, to improve balance. This neuromuscular strategy has been reported in patients with other neurological pathologies which cause postural impairment [8,9,10]. The function of the plantarflexors during SS is very important to support the body weight [39]. Moreover, the SS sub-phase is particularity unstable because all the bodyweight is supported on one limb. The results showed that during this sub-phase, the patients with the greatest magnitude of knee muscle coactivation were the most unstable. Moreover, SS sub-phase duration was reduced in these patients, as has been found in patients with postural instability during gait [38,39]. Furthermore, the reduction of SS duration was positively correlated with the reduction in magnitude of ankle muscle coactivation in the MA limb. Thus, postural instability during this sub-phase seems mainly due to insufficiency of the plantarflexor muscles (decreased coactivation between agonist and antagonist ankle muscles), which could be partly compensated for by an increase in coactivation of the quadriceps and hamstring muscles. This strategy could facilitate bodyweight support during the stance phase of gait, by the simultaneous production of a larger flexor moment at the hip and a larger extensor moment at the knee [9]. The reason why ankle muscle coactivation was reduced in the SS sub-phase in the MS patients is unclear. We hypothesize that in these patients, the efficiency of gait depends on adaptations of the neuromuscular system by the modulation of muscle activation in response to external demands that differ depending on the gait phase (DS or SS). Since only one limb is in contact with the ground during SS, ensuring stability is more challenging than in DS [40]. In consequence, the motor input to the ankle plantarflexor muscles maybe insufficient. To compensate, the neurological system might use different compensatory strategies, such as increasing the motor input of the knee muscles, less affected by the disease, to ensure stability. This hypothesis is in accordance with the results of Adam et al. (1990) [41] which showed that the ankle muscles are more affected by the disease than the knee muscles. The greater coactivation of the knee muscles may be related to spasticity of one or several heads of the quadriceps. However, the involvement of spasticity in the excessive coactivation of knee muscles is difficult to determine in this study, for the same reasons previously mentioned for the ankle muscles.

Overall, the results showed that the patients with MS adopted a “proximal” strategy during the SS sub-phase, involving increased knee muscle coactivation in the MA limb to stiffen the knee joint and improve stability during forward progression. However, the same strategy was not used in the LA limb since there were no significant correlations between the coactivation parameters of the LA knee muscles and the parameters relating to postural stability. Thus, the role of knee muscle coactivation in the LA lower limb to ensure postural stability during the SS sub-phase seemed minor.

## Limits

This study has several limitations. Firstly, the patients walked significantly more slowly than the healthy subjects. Since gait speed affects muscle coactivation [36], this could have led to interpretation bias. Secondly, the small number of patients and healthy subjects included could limit the statistical power of the results. Thirdly, the patients included had mild to moderate levels of disability with EDSS scores ranging from 2.0 to 5.0, and they had low levels of muscle spasticity. Further studies are thus required to investigate coactivation in patients with more severe disability (EDSS>5.0) and higher level of spasticity.

## Conclusion

Several studies have characterized the gait patterns of patients with MS [13,15,16,17,18] by analyzing alterations in kinematic parameters, timing and sEMG signal amplitudes [17,18]. The present study analysed the simultaneous activity of agonist and antagonist muscles during gait, demonstrating that abnormal lower limb muscle coactivation occurs during the stance phase of gait in patients with MS, even with mild impairments. Overall, the results suggest that the increased lower limb muscle coactivation during gait is an adaptive strategy, however it may also be related to spasticity. Moreover the results highlighted two distinct strategies: proximal and distal, based on limiting the number of degrees of freedom of a joint (ankle or knee), depending on the phase of the gait cycle, to reduce postural instability caused by muscle weakness. During the DS sub-phases, the patients used a “distal” strategy, involving increased coactivation of the ankle muscles. During the SS sub-phase, they used a “proximal” strategy with increased coactivation of the knee muscles. Although the pattern of muscle coactivation was similar in both lower limbs (MA and LA), the functional role of the coactivation differed between limbs. More particularly, coactivation in the MA limb seemed to play a large role in limiting postural instability throughout the whole gait cycle whereas coactivation in the LA lower limb appeared to play a very minor role in postural stability during stance.

We propose that for patients with MS who have abnormal levels of lower limb muscle coactivation during gait, rehabilitation programs should focus on balance exercises and dynamic muscle strengthening (mainly the ankle plantarflexors) of the MA lower limb. However, further studies are necessary to evaluate the effectiveness of these methods.
